# Magnetic Particle Imaging for Pulmonary Applications: Technological Advances, Biological Insights, and Clinical Translation

**DOI:** 10.3390/bioengineering13060635

**Published:** 2026-05-29

**Authors:** Shiva Toumaj, Ahmed Afifi, Muhiddin Dervis, Doaa Mashaly, Abdallah Abudraz, Abdulahi Hassan, Mohamad Rustm, Sachin Jambawalikar, Muhammad Umair

**Affiliations:** 1 Faculty of Medicine, Urmia University of Medical Sciences, Urmia 51369-67765, Iran; 2 Faculty of Medicine, Benha University, Benha 13518, Egypt; 3 Faculty of Medicine, Harvard Medical School, Massachusetts General Hospital, Boston, MA 02215, USA; dr.mohi.dar@gmail.com; 4 Faculty of Medicine, October 6 University, Giza 12585, Egypt; doaamashaly@gmail.com; 5 Faculty of Medicine, Ankara Yildrim Beyazit University, Ankara 06800, Turkey; abdullahdraz55@gmail.com (A.A.); aohassan404@gmail.com (A.H.); mhdrustm@gmail.com (M.R.); 6 Department of Radiology, Columbia University, New York, NY 10027, USA; sj2532@cumc.columbia.edu; 7 Department of Radiology, Columbia University Irving Medical Center, New York, NY 10027, USA; mu2331@cumc.columbia.edu; 8 Department of Radiology, Johns Hopkins University, Baltimore, MD 21218, USA

**Keywords:** magnetic particle imaging, superparamagnetic iron oxide nanoparticles, pulmonary imaging, hybrid imaging, functional lung imaging

## Abstract

Background: Magnetic particle imaging (MPI) is an emerging, tracer-based modality that directly detects superparamagnetic iron oxide nanoparticles (SPIONs) with exceptional sensitivity, quantitative signal behavior, and full immunity to air–tissue susceptibility artifacts. These features make MPI particularly well-suited for pulmonary imaging, where traditional techniques such as computed tomography (CT), magnetic resonance imaging (MRI), and nuclear medicine-based ventilation/perfusion (V/Q) imaging are limited by radiation exposure, low contrast, and motion-related signal degradation. Objective: This review synthesizes the current state of MPI for lung imaging, with emphasis on its physical principles, tracer development, preclinical applications, and its potential role in assessing pulmonary perfusion, vascular integrity, inflammation, and therapeutic responses. Methods: A systematic evaluation of preclinical studies was performed across three major application domains: pulmonary perfusion mapping, cell tracking and therapeutic monitoring, and vascular injury and permeability assessment. Study designs, SPION formulations, MPI acquisition strategies, and validation methods, including histopathology, biodistribution, broncho-alveolar lavage fluid (BALF) analysis, and Evans Blue assays, were examined to characterize methodological consistency and imaging performance. Results: MPI consistently demonstrated high-contrast, quantitative visualization of pulmonary blood flow, endothelial barrier disruption, inflammatory signaling, and transplanted or inhaled cell populations. Tracer engineering played a critical role: macroaggregated albumin superparamagnetic iron oxide nanoparticles (MAA-SPIONs) enabled capillary-level perfusion mapping, LS-008 improved temporal resolution and vascular delineation, Synomag/Synomag-D allowed quantification of vascular leakage in acute and chronic lung injury, and vascular cell adhesion molecule-1 (VCAM-1)-targeted probes provided molecular-level assessment of inflammation. Hybrid MPI-CT and MPI-MRI approaches further enhanced anatomic localization and enabled accurate pulmonary blood volume (PBV) estimation. Across studies, MPI measurements showed strong agreement with established biological assays and remained free of the artifacts that limit CT and MRI in the lung. Conclusions: Preclinical evidence demonstrates that MPI is a robust, radiation-free, and quantitatively precise modality for functional and molecular lung imaging. Its ability to map perfusion, track therapeutic agents, and noninvasively quantify vascular permeability positions MPI as a promising future alternative or complement to CT, MRI, and nuclear medicine for pulmonary assessment. Continued tracer optimization, system scaling, and clinical validation are key steps toward translating MPI into routine clinical use.

## 1. Introduction

Magnetic particle imaging (MPI) is an emerging tracer-based imaging modality that detects superparamagnetic iron oxide nanoparticles (SPIONs) exposed to oscillating magnetic fields and spatially selective magnetic gradients [1,2,3]. MPI generates high-contrast images without background noise as only the particles administered are the source of the signal. This approach has advantages of high temporal resolution, quantitative signals and avoids susceptibility artifacts at air–tissue interfaces. MPI avoids ionizing radiation, unlike computed tomography (CT) and nuclear imaging; moreover, it forgoes proton density and field homogeneity, differentiating it from magnetic resonance imaging (MRI). These features make MPI a unique modality for applications where traditional techniques face technical limitations [4].

Pulmonary imaging remains technically challenging due to the lung’s combination of low proton density, complex air–tissue interfaces, respiratory motion, dynamic vascular permeability, and heterogeneous regional perfusion. Each of these factors can complicate interpretation or reduce image quality, depending on the imaging modality used. CT and nuclear imaging can provide crucial information related to structure and function but are associated with ionizing radiation. Moreover, MRI may be affected by factors such as low signal intensity or signal loss in the lung parenchyma [4]. These limitations have driven interest in alternative imaging modalities that can provide quantitative assessment of pulmonary physiology without radiation exposure. MPI, therefore, appears to be a promising alternative in this context, allowing direct tracer detection with zero tissue background signal and immunity to air–tissue susceptibility artifacts [5,6].

MPI has been extended for pulmonary imaging to map ventilation, quantify perfusion, and assess vascular permeability in preclinical models [5,6,7]. SPION aerosols image airflow distribution; macroaggregated albumin superparamagnetic iron oxide nanoparticles (MAA-SPIONs) and Synomag^®^ (micromod Partikeltechnologie GmbH, Rostock, Germany) (small-core particles) track capillary perfusion and endothelial disruption intravenously. These studies demonstrate several potential advantages of MPI for lung imaging, including the absence of susceptibility artifacts at air–tissue boundaries, radiation-free functional assessment and the ability to generate quantitative high-contrast images without reliance on proton density or radioactive tracers. MPI uniquely balances safety, sensitivity, and real-time capabilities for pulmonary imaging, a profile unmatched by CT, MRI, or nuclear methods [8,9].

While MPI has shown growing promise in pulmonary imaging, most studies have focused on investigating specific applications such as ventilation, perfusion, or vascular permeability assessment in preclinical settings [5,6,7,8]. Although broader MPI reviews summarize the general principles and applications of the modality [1,9,10,11], a focused overview of pulmonary-specific MPI applications, associated challenges, and translational hurdles remains limited. This review addresses the gap by integrating current evidence across various pulmonary MPI applications within a lung-focused framework.

This review synthesizes the relevant physics for thoracic imaging, evaluates SPION formulations used in pulmonary applications, and summarizes key preclinical findings across ventilation, perfusion, and vascular health, thereby addressing this gap. In addition, this review discusses current translational barriers and future directions related to the clinical implementation of pulmonary MPI [12].

The main objectives of this review are:

- Emphasizing the physical fundamentals of MPI of relevance to pulmonary imaging.
- Discussing current SPION formulations for aerosolized and intravenous lung applications.
- Examining the important preclinical studies of ventilation, perfusion and vascular permeability.
- Discussing technical and translational challenges that need to be addressed to enable future clinical translation.

This review introduces MPI’s principles and physics and compares field-free line (FFL) and field-free point (FFP) encoding strategies that define system performance. MPI applications in pulmonary imaging span perfusion assessment, therapeutic cell tracking, drug delivery monitoring, and visualizing lung injury, fibrosis, and vascular permeability. The review integrates findings across studies by synthesizing methodological characteristics, quantitative performance metrics, biological signal behavior, and cross-study consistency. Finally, the discussion contextualizes these advances, outlines current limitations, and highlights future directions for advancing MPI as a quantitative tool for functional and molecular lung imaging.

## 2. Principles and Imaging Physics of MPI

MPI is a novel imaging modality that detects nonlinear magnetization from SPIONs subjected to static magnetic selection gradients and oscillating magnetic field driving forces, or more simply, a magnetic field gradient and an oscillating magnetic field. The signal is produced in the FFP, or FFL, where the SPIONs are not in the saturation state, producing higher-order harmonics. These harmonics are filtered and combined into high-resolution images. Additional sensitivity can be obtained using FFL-based systems [13]. Spatial encoding is performed by scanning the FFP over the picture area, either mechanically or using moving magnetic field gradients [14]. Eliminating Barkhausen noise and performing a complete harmonic analysis of the response to the driving field are two main ways to improve the MPI sensitivity and to improve the possibility to detect a linear relation between the signal intensity and the mass of the tracer. This allows us to capture images that are very detailed in both time and space [2,15].

The magnetic relaxation of SPIONs, according to Langevin statistics, is principally due to the Brownian rotation of the whole particle, and the Néel relaxation, the flipping of the internal magnetic moment. These two mechanisms respectively dominate at low frequencies (low driving field) and high frequencies. Optimizing particle size and driving field parameters can improve MPI signal, allowing use in low field settings in large organs, such as the lungs. The standard tracer material is the SPION Resovist^®^ (Bayer Schering Pharma AG, Berlin, Germany) (ferucarbotran), which has a hydrodynamic diameter of about 60 nm [16,17]. Though representing only 3% of the iron content [18,19], MPI images demonstrated real-time 3D imaging in small animals at frame rates of 50 volumes per second [15]. Tracer development continues; steps include the fabrication of SPIONs with controlled particle sizes and magnetic properties in order to achieve a better MPI signal [20]. Towards the upper end of the size range, MAA-SPIONs are used in MPI of pulmonary perfusion [5]. Smaller-core ferromagnetic nanoparticles, such as Synomag^®^, have shown promise as MPI tracers for evaluating pulmonary vascular abnormalities, including pulmonary embolism (PE), acute respiratory distress syndrome (ARDS), and pulmonary fibrosis (PF) [6,21]. This development expands the scope of MPI to functional and molecular imaging in preclinical settings.

## 3. Comparison of FFL and FFP Encoding in MPI

Spatially varying magnetic fields are used in MPI to create field-free zones in which the SPIONs can respond to an applied drive field, creating a signal. Two basic forms of MPI instrumentation use either the FFP or FFL encoding geometries which are described in the following sections. In FFP-based systems, a zero-field point is scanned through the sample volume, and only the nanoparticles at the FFP contribute to the signal at any given time. This leads to high spatial resolution and localized excitation of the sample. However, due to the sequential nature of the scanning of the FFP through many positions, FFP systems are usually slower and have a smaller effective field of view than line-based systems [22].

**Figure 1:**
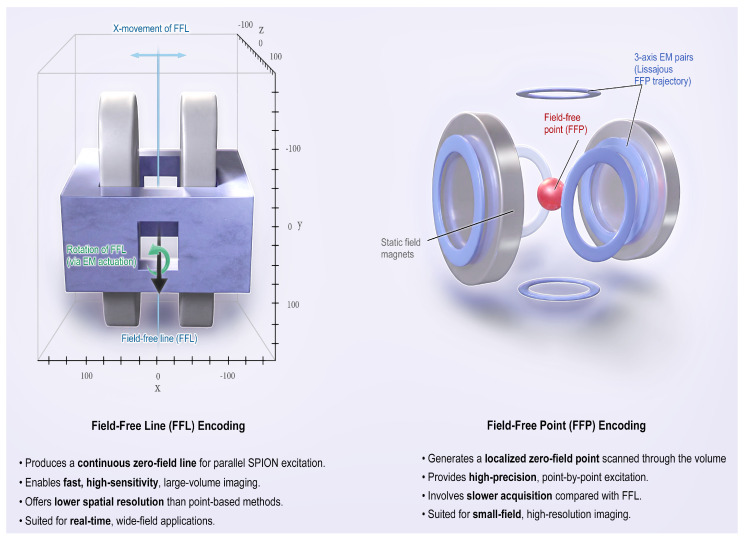
Schematic illustration of field-free point and field-free line configurations in magnetic particle imaging.

In FFL encoding, a continuous line of zero magnetic field can be generated to excite all SPIONs in a line. Since the line can be scanned over a large portion of the imaging volume, FFL systems can be very sensitive and are very useful for dynamic or real-time imaging of fast changing processes like hemodynamic changes and pulmonary perfusion. However, a disadvantage of FFL imaging is that it has poorer spatial resolution compared to FFP, since the signal is integrated along an entire line instead of at a single point [23]. The FFP and FFL strategies are complementary. FFP strategies can achieve high spatial resolution imaging for small structures or molecular imaging while FFL strategies can achieve faster imaging and higher sensitivity when imaging a large volume of the subject. Since many pulmonary imaging applications require high speed, high sensitivity and a large field of view, FFL strategies are commonly used for pulmonary imaging. The major differences between the FFP and FFL encoding are outlined in Figure 1.

## 4. Potential Applications of MPI in Pulmonary Imaging

The absence of a background signal in addition to the flexibility of this contrast agent has made MPI a modality of choice to assess a number of physiological and pathological processes of the lung. While it has been mainly used for perfusion measurements, other applications include ventilation assessment, cellular therapy tracking, vascular leak studies and inflammation quantification. These applications highlight MPI’s potential to overcome the limitations related to other imaging modalities, including radiation exposure, limited sensitivity, and susceptibility to artifacts due to the air-filled lung environment. The following subsections summarize the most interesting preclinical studies performed using MPI on the pulmonary vasculature, lung structural imaging, and evaluation of therapies.

### 4.1. Pulmonary Perfusion Imaging with MPI

Data on blood flow in major vessels and capillary-level perfusion are crucial for diagnosing and staging lung vascular disorders like PE. PE patients often present with non-specific symptoms, such as chest pain and shortness of breath, which makes diagnosing PE particularly difficult for clinicians [5,24]. Therefore, imaging studies are vital for an accurate differential diagnosis of PE. CT pulmonary angiography (CTPA) and ventilation/perfusion (V/Q) scans are the primary imaging methods used clinically to diagnose PE [25,26]. MRI is less commonly used due to its poor diagnostic performance in lung parenchymal imaging, although magnetic resonance angiography (MRA) of the pulmonary artery is an established approach for pulmonary artery pathologies including PE [27,28,29]. V/Q scans are often limited to availability during regular weekday hours; furthermore, the need for immediate preparation of 99mTc albumin aggregated (99mTc-MAA) before the scan can lead to wait times of over two hours [26,30]. V/Q scans are preferred for patients with chronic kidney disease (CKD) or contrast allergies [31]. In contrast, CTPA is utilized more in clinical practice due to its rapid and high-resolution imaging capabilities, although it involves higher radiation exposure and poses risks to CKD patients or those with contrast allergies [32,33].

MPI is known for its high sensitivity and excellent image contrast, as background tissues like bone, muscle, blood, and fat produce no MPI signal, and low-frequency magnetic fields penetrate tissue without depth attenuation. However, a key limitation of MPI in pulmonary imaging is that it primarily visualizes the tracer signal within the pulmonary vasculature; it does not provide direct structural imaging of the lung parenchyma or airways themselves. This has prevented its translation into the clinic aside from vascular-based processes and diseases [5,34]. Comparatively, the advantages of MPI also address many of the shortcomings associated with the existing standard imaging modalities for PE, including the need for rapid, non-intrusive imaging that can visualize pulmonary blood flow without the use of ionizing radiation. The pulmonary vasculature is a favorable target for imaging. Unlike MRI, the low homogeneity requirement of the drive field ensures that the MPI signal in the pulmonary capillaries is not affected by the ±5 ppm field perturbation from surrounding air-filled alveoli. Therefore, MPI may not be affected by the majority of the artifacts that plague conventional MRI. Additionally, MPI does not utilize ionizing radiation or contrast agents, unlike CT. MPI particles, unlike tracers used in V/Q scans, do not quickly degrade following preparation before administration [5]. Furthermore, the ability of MPI to generate purely quantitative maps of tracer distribution provides a functional assessment of pulmonary perfusion that is inherently linear with iron concentration. This quantitative nature makes MPI particularly suitable for evaluating subtle regional perfusion deficits that may be missed by standard imaging modalities. Given that perfusion defects are often the earliest indicators of pulmonary vascular disease, even before structural abnormalities become visible, MPI has the potential to complement or enhance current PE and microvascular disease diagnostics.

In 2017, Zhou, Jeffris, Elaine, Zheng, Goodwill, Nahid and Conolly [5] were the first to produce high-contrast in vivo MPI lung perfusion images in rats using a novel lung perfusion agent, MAA-SPIONs, and evaluated the feasibility of using these images to diagnose PE safely and accurately [5]. They hypothesized that because the lung capillary diameter is smaller than the MAA-SPION particle size, an intravenous bolus of MAA-SPIONs would be trapped in the lungs, facilitating lung imaging [35,36]. Conversely, non-labeled SPIONs, being smaller than the lung capillary diameter, would bypass the pulmonary vasculature and accumulate in the liver and spleen. To confirm this hypothesis, Zhou, Jeffris, Elaine, Zheng, Goodwill, Nahid and Conolly [5] tail-vein-injected three groups of rats with either a Perimag^®^ (micromod Partikeltechnologie GmbH, Rostock, Germany) (SPION) bolus, a Perimag bolus with MAA, or a saline bolus, and then performed a CT scan directly 10 minutes after injection. The animals were then sacrificed and dissected, and the lungs, liver, heart and spleen were imaged with an MPI system to show the location of the tracer [5]. The study demonstrated that MAA-SPIONs were detectable in MPI and successfully targeted lung perfusion within 10 minutes post-venous injection, whereas SPIONs alone failed to target the lung and were instead cleared to the liver and spleen within the same timeframe. This substantiated the hypothesis that lungs would accumulate MAA-SPIONs subsequent to intravenous administration. They created 3D images of lung perfusion that were very sensitive and very clear. This suggests that MPI might be a good way to accurately and safely diagnose life-threatening lung conditions like PE [5]. These results also set up an early framework for functional perfusion imaging based on MPI. Later studies built on this by adding hybrid systems, advanced tracers, and dynamic imaging strategies [34]. As MPI technology improves, the creation of specialized perfusion tracers, like MAA-SPIONs made for capillary trapping, increases the probability that MPI is a good way to look at the pulmonary vascular system.

Investigations have explored supplementary potential applications of MPI in pulmonary imaging, including the assessment of pulmonary blood volume (PBV). PBV is the amount of blood in the pulmonary blood vessels at any time in a resting state [37]. PBV is an important hemodynamic parameter to study because it is affected by both normal and abnormal physiological processes. PBV is based on cardiac output and the ability of the pulmonary vasculature to stretch, which gives us useful information about pathological processes that change these parameters [38]. Recent clinical studies have demonstrated that PBV functions as a prognostic indicator for diseases impacting the heart and lungs, specifically chronic heart failure and acute PE [39,40]. PBV could also be used as a parameter in preclinical research on cardiovascular and pulmonary conditions. Nonetheless, the translation of PBV evaluations to small-animal models necessitates imaging techniques that can accurately capture extremely rapid cardiopulmonary dynamics while minimizing motion-induced artifacts. Noninvasive measurement of PBV in mice poses challenges due to their small size and high heart rate [38].

Different ways have been created to measure PBV. At first, the indicator dilution technique meant putting dyes into the heart chambers and pulmonary circulation [41]. Later, nuclear radiography made it possible to follow injected radioisotopes in the heart [42]. MRI and MRA facilitated the evaluation of cardiac function and the tracking of contrast agent boluses within the cardiopulmonary vasculature without the use of ionizing radiation [43]; however, the tracking of boluses is qualitative and not quantitative [38]. These problems made it clear that we needed an imaging strategy that could combine the best features of different modalities to get both detailed images of anatomy and fast-moving tracers.

In 2017, Kaul, et al. [44] examined the use of a new monodisperse tracer called LS-008 and compared it to the standard tracer Resovist. They conducted both in vitro and in vivo MPI measurements, encompassing magnetic particle spectroscopy (MPS), phantom studies, and dynamic scans in healthy friend virus B-type (FVB) mice. MRI was done first to get an anatomical reference, and then LS-008 and Resovist were injected at the same volume and concentration in two different settings. In vitro, LS-008 showed higher sensitivity, and MPI demonstrated improved vessel delineation, resolving a larger number of vessels and allowing for distinction of the aorta from the vena cava, which was not possible with Resovist. Following intravenous injection, both tracers showed bolus movement through the inferior vena cava in vivo, but LS-008 showed fewer temporal artifacts and detectable pulsation due to respiratory and cardiac cycles. LS-008 allowed clear distinction of the aorta from the IVC and revealed additional liver vessels not seen with Resovist. The blood half-life of LS-008 was 88 minutes, compared to 13 minutes for Resovist, which accumulated rapidly in the liver. LS-008 also enabled measurement of liver and kidney perfusion fractions. These early findings established LS-008 as a superior blood-pool tracer for MPI, motivating further investigation into its utility for cardiopulmonary functional imaging.

Building on this tracer optimization work, the same group later applied MPI in a hybrid configuration to enable quantitative cardiopulmonary assessment, including PBV measurement. Kaul, Mummert, Graeser, Salamon, Jung, Tahir, Ittrich, Adam and Peldschus [38] aimed to overcome these limitations by combining two imaging modalities to determine PBV in mice. In 2021, they investigated PBV in mice by leveraging the high temporal resolution of MPI and the high spatial resolution of MRI to analyze the passage of SPIONs from the right ventricle through the lungs to the left ventricle, as well as to determine cardiac function. It is crucial to accurately measure PBV in order to understand how blood flows in the pulmonary system and to find small problems with the microvasculature. This makes this hybrid method very useful. Eight FVB mice underwent MRI scans utilizing a volumetric body coil, yielding high-resolution anatomical images of the cardiac structures. After the MRI scans, a small amount of a highly concentrated tracer was injected along with saline during MPI assessments. This made it possible to accurately measure the pulmonary transit time (PTT) and the RR interval length. The MRI and MPI images were aligned and combined, successfully showing the SPION bolus moving through the heart and pulmonary vessels. This displayed the timings of the tracer’s arrival in the left ventricle, right ventricle, and pulmonary vasculature. By evaluating stroke volume, PTT, and RR interval duration, the group calculated PBV. This suggested that PBV could be accurately calculated with a narrow margin of error using the combined MPI-MRI approach [38]. Together, these studies demonstrate how improved tracers and multimodal imaging can improve MPI’s functionality from simple perfusion visualization to a comprehensive cardiopulmonary functional assessment.

Preclinical research has repeatedly demonstrated that MPI can evaluate cardiopulmonary hemodynamics with high sensitivity and properly represent pulmonary perfusion, as shown in Table 1. Zhou, Jeffris, Elaine, Zheng, Goodwill, Nahid and Conolly [5] demonstrated that lung perfusion imaging with MAA-SPIONs is possible in principle. This was the first in vivo proof that MPI can accurately show perfusion defects. MAA-based tracers temporarily lodge in the pulmonary microvasculature in proportion to regional blood flow. Regions of decreased or absent signal correspond to perfusion defects, supporting applications in PE, pulmonary infarction, and chronic thromboembolic pulmonary hypertension (CTEPH). Further research by Kaul, Mummert, Jung, Salamon, Khandhar, Ferguson, Kemp, Ittrich, Krishnan and Adam [44] revealed how useful optimized tracers like LS-008 could be. These tracers made vascular contrast, temporal resolution, and circulation time significantly better than standard agents. More recently, hybrid MPI-MRI methods have made it possible to accurately and noninvasively estimate PBV by combining MPI’s fast bolus tracking with MRI-derived cardiac volumetry. Together, these studies show that MPI is becoming a more useful tool for measuring the function of the pulmonary vasculature without using radiation.

Despite promising perfusion imaging results, most studies remain limited to proof-of-concept animal experiments using non-clinically approved tracers. Future work should focus on clinically translatable iron oxide agents and standardized quantitative perfusion metrics to facilitate translation into human pulmonary imaging.

### 4.2. Lung Cell Tracking and Therapeutic Monitoring with MPI

MPI has emerged as a powerful modality for tracking therapeutic agents and cellular therapies in the lung due to its high sensitivity, quantitative signal response, and complete absence of background noise [45]. Lung diseases such as PF, chronic lung injury, and obstructive airway disorders have shown some utility of cell-based therapeutic strategies, yet monitoring the fate of transplanted or inhaled cells remains a major challenge. Traditional imaging modalities, including MRI and single-photon emission computed tomography (SPECT), face limitations such as low sensitivity, signal interference from surrounding tissues, radiation exposure, or the need for complex radiolabeling procedures. By employing SPIONs as tracers, MPI, on the other hand, gets over these obstacles and provides a noninvasive, high-contrast imaging method that is especially well-suited for long-term lung-targeted treatment monitoring.

This capacity was examined by Nigam, et al. [46] in the setting of PF, a highly morbid and progressive disease marked by decreased oxygen exchange, extracellular matrix accumulation, and recurrent epithelium damage. Cell therapy has drawn interest as a potential therapeutic approach because of the limited availability of treatment alternatives, which often just slow the progression of the disease. However, the capacity to monitor the distribution, survival, and retention of transplanted cells over time is crucial to the effectiveness of such treatment. In order to follow human distal lung epithelial progenitor cells administered intratracheally into immunocompromised mice, Nigam and colleagues assessed MPI. Immunostaining and iron quantification tests were used to verify the labeling effectiveness of the SPIONs used to label the transplanted cells. The initial distribution of transplanted cells and their persistence for up to two weeks could be clearly seen thanks to MPI. Gradual cell loss or migration was indicated by a progressive ~65% decrease in signal intensity, and histological analysis validated the imaging findings. This study demonstrated that MPI provides a reliable and quantitative method for in vivo tracking of therapeutic lung cell populations, allowing longitudinal assessment that is difficult to achieve with conventional imaging modalities.

MPI has a lot of potential for monitoring inhaled therapeutic agents in addition to cell transplantation treatment. According to Tay, Chandrasekharan, Zhou, Yu, Zheng and Conolly [7], the noninvasiveness, rapid systemic uptake, and direct targeting of the lung make pulmonary drug administration clinically attractive. However, the practicality of traditional imaging methods like SPECT is limited by the need for short-lived radiotracers and complex synthesis steps before each scan. They showed that MPI can track SPION-labeled aerosols in vivo without the use of radiochemistry. Their proof-of-concept studies evaluated aerosol deposition, delivery efficiency, and dispersion patterns, providing detailed insight into initial therapeutic distribution within the lungs. Crucially, MPI provided a useful tool for studying the clearance dynamics of inhaled therapeutics by visualizing the entire mucociliary clearance pathway, from initial deposition in the bronchial tree to transport toward the epiglottis and eventual passage into the gastrointestinal tract.

Additionally, Tay, Chandrasekharan, Zhou, Yu, Zheng and Conolly [7] highlighted more extensive therapeutic options. Susceptibility artifacts and low sensitivity limited previous attempts to track SPION-based aerosols using MRI; however, MPI’s linear signal response and metal-specific contrast overcome these issues. Additionally, the quantitative nature of MPI allows researchers to assess both spatial distribution and dose delivery of therapeutics accurately. According to the authors, MPI might be used with cutting-edge therapeutic strategies including targeted delivery, magnetic actuation, or treatments based on hyperthermia. Through these applications, MPI’s clinical and preclinical significance is extended from diagnostics to active therapeutic guidance. Together, these studies establish MPI as a transformative imaging modality for lung therapeutic monitoring. MPI offers unparalleled sensitivity, real-time capabilities, and quantitative assessment, whether tracking transplanted progenitor cells for regenerative therapy in PF or visualizing inhaled nanoparticles for drug delivery optimization. The approach is positioned to play a key role in the development, assessment, and ultimate clinical translation of lung-targeted treatment options as SPION engineering and MPI system performance continue to progress.

Beyond regenerative and therapeutic monitoring applications, the ability of MPI to quantitatively track transplanted or targeted cell populations may also provide important opportunities in lung oncology. Real-time visualization of cellular distribution and retention could support evaluation of tumor-targeted therapies, immune-cell trafficking, and nanoparticle-mediated therapeutic delivery within the pulmonary microenvironment. These capabilities may facilitate future development of personalized therapeutic strategies and longitudinal monitoring approaches in pulmonary malignancies.

An overview of important studies that have used MPI to track therapeutic cells and inhaled agents within the lung is provided in Table 2. Nigam, Uhl, Bamrah, Lin, Jager, Lawson, Girgis, Kenyon, Li and Li [46] utilized MPI to monitor SPION-labeled distal lung epithelial progenitor cells in fibrotic mice, demonstrating the technique’s ability to quantify cellular persistence and spatial distribution over two weeks. Tay, Chandrasekharan, Zhou, Yu, Zheng and Conolly [7], on the other hand, concentrated on aerosol-based therapies, demonstrating that MPI can dynamically detect deposition patterns and mucociliary clearance pathways with high sensitivity. Together, these findings underscore MPI’s versatility in evaluating both regenerative cell therapies and pulmonary drug delivery strategies.

Despite MPI’s robust ability to monitor therapeutic cells, problems persist due to variability in labeling efficiency, tracer clearance, and the lack of standardized quantification methodologies. Subsequent research should concentrate on clinically validated tracers and longitudinal verification in disease-specific models.

### 4.3. MPI for Lung Injury, Fibrosis, and Vascular Permeability

Researchers have investigated MPI’s ability to evaluate pathological changes in lung vascular integrity by utilizing its established capabilities in perfusion and cell tracking. MPI can reveal information about the progression of diseases including chronic fibrosis and acute lung injury (ALI) by identifying subtle vascular abnormalities. In order to evaluate pulmonary vascular permeability and leakage, Feng, Gao, Li, Hui, Jiang, Xie and Tian [6] investigated MPI using small SPION particles that penetrate the capillary–endothelial barrier in diseased situations [6]. The pulmonary endothelium normally functions as a strong barrier to stop blood components from seeping into lung tissue. During ALI and chronic lung diseases, this barrier is disrupted, causing increased vascular permeability, which contributes to mortality in ARDS and poor prognosis in PF. These clinical challenges underscore the need for imaging techniques that can noninvasively monitor vascular integrity and dynamics over time. Therefore, there is a need for techniques to visualize and quantify changes in pulmonary vascular integrity in vivo throughout disease progression [47,48,49,50,51].

In addition to vascular leakage assessment, MPI has also been applied to detect and quantify intra-alveolar inflammation, a key pathological feature of acute ALI. Gao, et al. [52] investigated intra-alveolar inflammation, a key feature and prognostic marker of ALI, which was difficult to visualize using conventional imaging. They aimed to develop a small-sized vascular cell adhesion molecule-1 (VCAM-1)-targeted MPI nanoprobe (ESPVPN) for molecular-level assessment of lung inflammation. ESPVPN was engineered by conjugating a peptide onto a polydopamine-coated superparamagnetic iron oxide core. Its MPI performance, targeting ability, and biosafety were characterized in vitro and in vivo. VCAM-1 expression was measured in human umbilical vein endothelial cells (HUVECs) and lipopolysaccharide (LPS)-induced ALI mouse models via Western blot and histopathology. Lung tissue inflammation and pro-inflammatory markers were quantified using enzyme-linked immunosorbent assay (ELISA). ESPVPN (~10 nm) demonstrated superior MPI performance compared to commercial tracers and showed effective targeting and safety. VCAM-1 expression correlated positively with LPS dose (R = 0.9381) and MPI signal (R = 0.9186), as well as with inflammatory markers (R > 0.7). The study concluded that ESPVPN effectively visualized and quantified lung inflammation in ALI models using MPI.

In research, pulmonary vascular permeability is typically assessed using terminal assays like the Evans Blue test and broncho-alveolar lavage fluid (BALF) total protein test [53]. These methods provide quantitative data but cannot track dynamic changes in the same animal or spatial distribution, treating the entire lung as a whole, producing readings that lack sufficient detail. This limitation hinders understanding of the spatial and temporal variations in endothelial dysfunction seen in diseases like PF and lung cancer [6]. While CT imaging is commonly used in pulmonary imaging to detect structural abnormalities and pulmonary edema, it cannot distinguish between hydrostatic pressure edema and permeability edema, even though only the latter is linked to vascular permeability [54,55,56]. There is currently a paucity of in vivo imaging techniques specifically targeting pulmonary vascular disruption, and MPI has the potential to revolutionize the measurement of pulmonary vascular permeability [6].

Previous studies have shown that nanoparticles with hydrodynamic sizes greater than 30 nm have negligible penetration through the endothelium [7]. Feng, Gao, Li, Hui, Jiang, Xie and Tian [6] hypothesized that Synomag particles, with an average core size of approximately 20 nm, would not be able to pass through a healthy endothelium. On the other hand, in disease states where the blood–lung barrier is disrupted, circulating nanoparticles can pass through the endothelium and extravasate into the alveolar space which would be detectable by MPI. Therefore, Synomag particles will only leak into lung tissue where the endothelial barrier is compromised.

Based on the aforementioned findings, Feng, Gao, Li, Hui, Jiang, Xie and Tian [6] investigated the feasibility of utilizing MPI signals to visualize and quantify pulmonary vascular leakage in both acute and chronic conditions. Because MPI signal intensity correlates linearly with iron concentration, the study demonstrated that it was possible not only to visualize but also to quantify pulmonary vascular leakage by injecting SPIONs and imaging the signals produced from diseased mice lungs, then comparing them with those of healthy lungs. MPI projection images were reconstructed and co-registered with CT images to obtain anatomical information. The pulmonary MPI signal in healthy mice was close to zero, whereas the pulmonary MPI signal in mice with ALI and PF was markedly increased, indicating vascular leakage. Feng, Gao, Li, Hui, Jiang, Xie and Tian [6] demonstrated that 3D MPI-CT successfully visualized and quantified pulmonary vascular disruption in vivo in both acutely injured and fibrotic mice lungs [6].

Building on this initial demonstration of MPI for permeability assessment, a subsequent study refined this approach for dynamic quantification of acute injury. Feng [57] studied pulmonary vascular permeability, a key feature of ALI and some chronic lung diseases. They pointed out that current techniques, including the Evans Blue test, lung wet/dry ratio, and BALF protein measurement, were unable to track dynamic vascular changes in vivo. They used quantitative image analysis, CT, and MPI in their study to noninvasively visualize pulmonary vascular permeability in animal models. Imaging experiments were conducted using an ARDS model induced by oleic acid (OA). In order to measure vascular permeability, they developed a pulmonary SPION extravasation index (SEI) based on 3D MPI-CT images. Ex vivo imaging results were in great agreement with the markedly elevated SEI observed in ARDS mice. Hematoxylin and eosin (H&E) histology, lung wet/dry ratio, BALF protein levels, and the Evans Blue assay were used to validate quantitative MPI-CT results. The study showed that 3D MPI-CT could track dynamic injury changes in vivo and assess pulmonary vascular permeability.

These studies collectively demonstrate the versatility of MPI for assessing pulmonary vascular integrity, permeability, and inflammation in preclinical models. Key preclinical MPI studies in this field are summarized in Table 3, which highlights the tracer type, experimental approach, and main conclusions. The table shows how consistently MPI can measure vascular leakage, identify small endothelial disruptions, and track dynamic inflammatory changes in vivo. These studies offer a thorough framework for noninvasive evaluation of lung injury and fibrosis by combining MPI with CT or molecular targeting techniques, highlighting MPI’s potential as a potent tool for mechanistic research as well as potential translational applications in pulmonary disease.

Current research mostly relies on experimental nanoparticle formulations, despite MPI’s high sensitivity for identifying vascular leakage. Clinical translation will require confirming permeability measurements across several lung injury models and assessing clinically approved iron oxide tracers.

MPI’s high sensitivity, superior contrast, and lack of ionizing radiation and air–tissue artifacts make it a promising tool for pulmonary imaging, particularly of the vasculature. MPI is a promising method for detecting PE and researching pulmonary vascular integrity since studies by Zhou, Jeffris, Elaine, Zheng, Goodwill, Nahid and Conolly [5] and Feng, Gao, Li, Hui, Jiang, Xie and Tian [6] have demonstrated its efficacy in imaging and measuring lung perfusion and vascular permeability. Furthermore, Kaul, Mummert, Graeser, Salamon, Jung, Tahir, Ittrich, Adam and Peldschus [38] revealed that the integration of MPI with MRI enhances PBV measurements, yielding significant insights for both clinical and preclinical studies [5,6,38].

## 5. Results

To put the results from this review into perspective, we briefly describe the methodological aspects of the studies included in this review such as the design of the tracers, imaging protocols, animal models, and validation strategies. This is critical to understanding how MPI performs in a wide range of pulmonary imaging applications, including perfusion imaging, vascular permeability, cellular tracking and imaging inflammation at the molecular level. The following sections can then be seen to better synthesize the technical capabilities, biological responsiveness and cross-study consistency of MPI, when the experimental frameworks that determine the data acquisition and analysis are first examined.

### 5.1. Study and Methodological Characteristics

**Figure 2:**
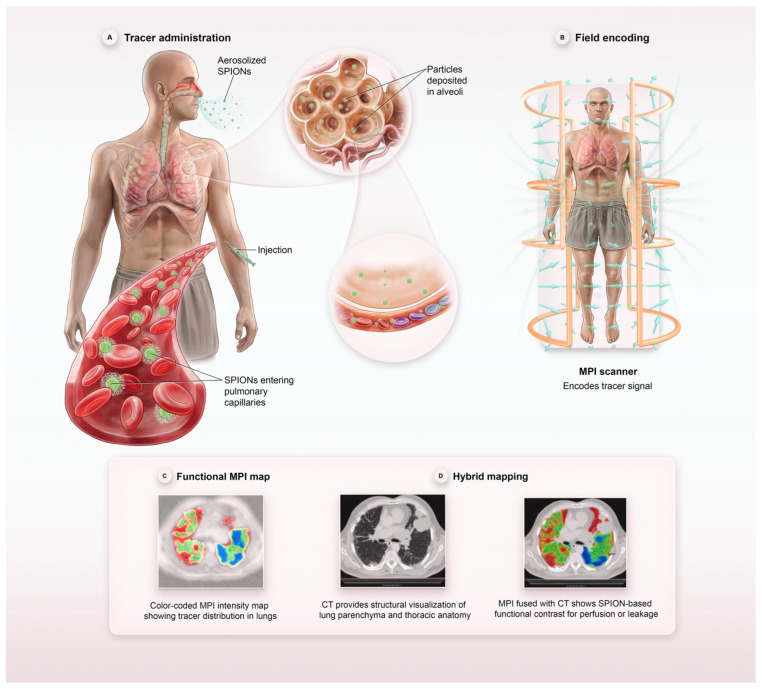
Pulmonary MPI workflow. (**A**) Tracer administration strategies, including inhalation of aerosolized SPIONs for alveolar deposition (**top**) and intravenous injection for capillary perfusion (**bottom**). (**B**) Magnetic field encoding where the scanner uses gradient fields to localize the tracer signal. (**C**) Functional MPI map showing a color-coded tracer intensity distribution in the lungs. (**D**) Hybrid mapping demonstrating structural lung anatomy via CT (**left**) co-registered with the functional MPI contrast (**right**) to pinpoint perfusion or vascular leakage.

The versatility of MPI in both functional and molecular imaging tasks was reflected in its use in the three experimental domains found across all of the literature reviewed, namely pulmonary vascular assessment, therapeutic monitoring, and characterization of pulmonary injury. The majority of investigations were performed in the context of small animal models (rats and mice), where the physiology of the lungs could be controlled and sufficient sensitivity for the detection of tracer dynamics based on SPIONs was available. Although the various disease models and imaging goals did not match exactly, the methodological frameworks were comparable in many ways, allowing the data from the various studies to be compared. Figure 2 illustrates the pulmonary MPI workflow, which consists of delivery of the tracer, encoding in the magnetic field, reconstruction of the images and merging of the CT-MPI images.

A variety of SPION formulations, targeting specific biological processes, were employed. To select perfusion imaging in the pulmonary microvasculature, aggregated particles containing MAA-SPIONs were designed to be mechanically trapped in the microvasculature [13]. Monodisperse blood-pool agents, such as LS-008, had a longer circulation time and increased magnetic responsiveness, and could be used for dynamic hemodynamic tests and hybrid imaging applications [42]. Two types of nanoparticles, Synomag and Synomag-D, containing smaller core sizes, were shown to be suitable for passive extravasation in disease situations in which the vascular barrier is compromised, as used in studies of endothelial barrier dysfunction [46,57]. To visualize inflammatory activity with high specificity at a molecular level, probes targeting activated endothelium like VCAM-1 targeted ESPVPN nanoparticle were developed [52]. The variety of the tracers emphasizes an ongoing trend of purpose-designed SPION agents to be used in specific pulmonary pathologies.

Studies reviewed in this report used both standalone and hybrid imaging platforms, most often MPI combined with either CT or MRI, to augment the quantitative signal of MPI with anatomical reference structures. Hybrid systems allowed for spatial co-registration of MPI signals with structural lung features, precise estimation of cardiac and pulmonary volumes and enhanced interpretation of perfusion and permeability data. The imaging protocols were different in terms of temporal resolution and tracer dose as well as in terms of acquisition configuration, but all were designed to maximize the sensitivity of SPION detection while minimizing artifacts due to respiratory motion, which is a major challenge in lung imaging.

The numerous ex vivo validation techniques were well integrated to guarantee biological validity. These were histopathologic scoring, biodistribution imaging, iron content quantification, analysis of BALF proteins, Evans Blue assays and wet/dry lung ratios. These techniques were used as standalone tests of MPIs and could be used to validate the permeability indices, inflammatory signals, cell persistence measurements, and perfusion patterns. This regularity of using the multimodal validation boosts the methodological strength of MPI research in pulmonary applications.

### 5.2. Imaging Performance and Quantitative Measurement Capability

Evaluation of the technical performance of MPI indicated a series of imaging characteristics that were both very desirable for pulmonary applications, especially the imaging response properties and quantitative fidelity. A prominent characteristic of MPI in all the studies was the degree of linearity between the concentration of iron in the sample and the signal intensity, allowing accurate estimation of the distribution of the tracer without being affected by the heterogeneity of the lung tissue. The background signal was not from bone, blood or air and was not affected by magnetic susceptibility gradients, so that the quality of the MPI signal did not degrade in regions in which MRI and CT often experience such degradation.

The magnetic properties and the pharmacokinetics of the MRI tracers were a major factor influencing the quantitative performance of MPI. Early generation agents, like Resovist, had short circulation times and were more prone to artifacts due to motion, and were therefore not useful for dynamic imaging. In comparison, LS-008, which is optimized for MPI, had an intravascular half-life of approximately 88 minutes, which is significantly longer than that for Resovist (13 min) [42]. The longer the blood-pool residence time contributed to bolus tracking stability, the more likely it was there would be a decrease in temporal noise, and the better the discrimination between the pulmonary and cardiovascular structures. The engineering of the tracers directly benefited the performance of MPI with the enhanced magnetization response and a smaller size distribution of the tracer LS-008 which led to higher spatial and temporal resolution.

**Figure 3:**
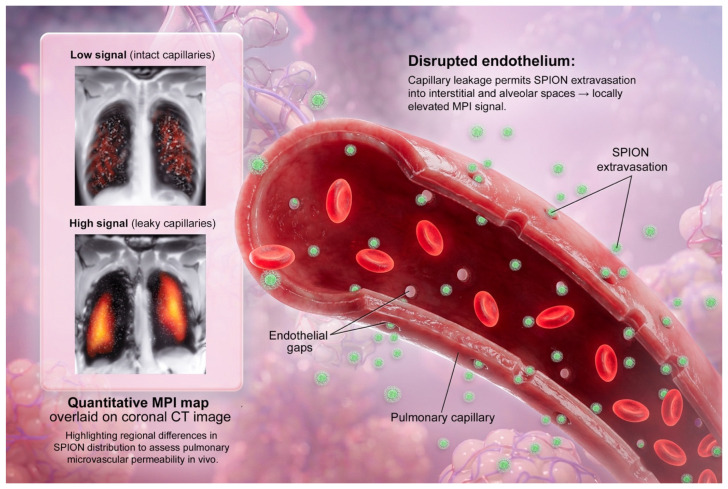
Pulmonary vascular permeability visualized using MPI, showing low signal in intact capillaries and elevated signal with SPION extravasation due to endothelial disruption; the quantitative MPI-CT overlay is an artist’s rendering.

During permeability studies, Synomag nanoparticles also offered additional proof of the quantitative precision of MPI. Synomag and Synomag-D only showed a measurable extravascular signal in models with endothelial barrier disruption (ALI and PF), but not in healthy lungs [46,57]. This brought high specificity for MPI to differentiate between intact and compromised areas. Furthermore, the intensity of the MPI signal was directly proportional to the severity of the vascular leak as reflected by good correlation with reference measurements such as determination of Evans Blue, BALF protein and wet/dry lung ratio. These results show that MPI is able to identify and quantify physiologically relevant changes in the integrity of the vascularity. SPION extravasation is depicted conceptually in Figure 3 together with the corresponding elevation of the MPI signal due to pulmonic microvascular leakage.

Hybrid imaging techniques also further enhanced MPI’s quantitative skills. To combine the temporal resolution of MPI with the anatomical and volumetric capabilities of MRI, protocols were developed in MPI that allow for accurate determination of PBV using transit times derived from MRI and chamber volume derived from MPI [36]. The reliability of these hybrid measurements with the already validated physiological ones validate the methodological strength of the combination of MPI and complementary modalities.

Given these results, the evidence shows that MPI offers a very robust, quantitative imaging platform for the assessment of pulmonary physiology, which can be improved upon by developing optimal imaging strategies and optimizing tracer formulation.

### 5.3. Functional and Biological Signal Behavior Across Models

MPI was consistently able to quantify changes in pulmonary physiology in a manner of biological significance in all of the vascular, cellular, perfusion, and molecular imaging studies. In models of ARDS, MPI-CT revealed over a twofold-higher permeability index compared to normal controls, and correlated well with Evans Blue accumulation, BALF protein levels, and lung wet/dry ratio [57]. Likewise, MPI was able to effectively track the therapeutic cell populations longitudinally and showed a decrease of about 65% in the signal associated with progenitor cells over 14 days, consistent with the histologic cell retention data [43].

Another set of perfusion-based investigations showed that MPI is sensitive to the tracer kinetics, with the lung-targeted agents generating immediate and high-contrast intravascular signal and the non-targeted SPIONs rapidly moving to the liver and spleen [13]. Molecular-targeted imaging was also found to be very accurate in terms of quantitative uptake, as the uptake of a VCAM-1 specific nanoprobe in ALI models correlated well with VCAM-1 expression (r = 0.9186) and various inflammatory biomarkers (r > 0.7) [52]. Taken together, these findings suggest that the use of MPI can provide high-precision quantitative information of dynamic physiological and pathological processes, thus supporting its use as a quantitative modality in pulmonary research.

### 5.4. Cross-Study Consistency and Strength of Evidence

Although the species and disease models used, the type of tracer (SPION) and the type of hardware varied among the studies, MPI performance was remarkably uniform throughout the literature. In all studies reported, they did not produce any artifacts and their MPI signal did not change when the MRI was taken in the presence of air–tissue interfaces that are known to degrade MRI quality. High reproducibility was also obtained for quantitative measurements obtained from MPI, which was also well in concordance with other reference methods, such as histopathology, Evans Blue assays, protein quantification in BALF and biodistribution imaging.

This uniformity was found at multiple points of the biological targets. MPI has proven to be sensitive and reliable in both acute and chronic lung disease models in relation to vascular leakage, inflammatory signaling, cell persistence and perfusion dynamics. Further, the development of tracer engineering has greatly enhanced imaging performance where LS-008 offered excellent temporal stability, Synomag-D was used to detect endothelial disruption and the ESPVPN nanoprobe was used for high molecular specificity. In combination, these results suggest that MPI is a well-developed and well-validated imaging modality for pulmonary imaging research that is both technically sophisticated and facile and is quantifiable with high accuracy and precision, while at the same time being biologically responsive.

## 6. Future Perspectives

Several areas of future research are needed to aid clinical translation of pulmonary MPI. Optimization of the size distribution, circulation time and targeting ability of the SPION-based tracers is crucial to enhance imaging sensitivity and specificity. Moreover, improvements in human-scale MRI scanners to incorporate better gradient design and motion compensation techniques are needed to allow whole-lung imaging.

Hybrid imaging techniques, e.g., MPI-CT and MPI-MRI, will also likely be important, since they will integrate functional MPI data with high-resolution anatomical reference images. In addition, there are studies that will assess the progression of disease, therapeutic monitoring and quantitative perfusion assessment in the longitudinal direction that will contribute to the standardization of imaging protocols. Lastly, future clinical trials that compare MPI to standard imaging techniques are needed to establish the diagnostic usefulness, safety and application of MPI in the management of pulmonary diseases.

## 7. Discussion

The advantages of MPI are that it is inherently insensitive to background tissue signal and susceptibility artifacts, and is not susceptible to susceptibility artifacts, which may affect image quality in traditional modalities like MRI or CT especially at air/tissue interfaces [4]. Because of these properties, MPI has a great advantage for pulmonary imaging, which is very complex. MPI is a safer option for individuals who have an increased risk of injury since it does not use damaging radiation or agents that could cause kidney damage. It delivers functional data at a high resolution without exposing people to radiation [2].

Although promising preclinical results have been obtained, there is an obvious translational gap between preclinical studies of pulmonary MPI and its clinical use. The majority of reported investigations involve the use of custom-designed SPIONs (MAA-SPIONs, LS-008, Synomag and targeted nanoparticles) which are not approved for human use. Conversely, there are clinically approved iron oxide agents with established safety profiles (ferumoxytol) which have yet to be studied in pulmonary MPI. This gap indicates an important research gap from tracer development to clinical translation. Pulmonary MPI should be studied in the future using clinically translatable or FDA-approved iron oxide formulations to shorten the process of regulatory approval and allow clinical studies in humans. Moreover, there is a need for standardization of imaging protocols, platform independence and direct comparison with clinical imaging modalities to move beyond proof-of-concept experiments to real-world pulmonary imaging applications.

In recent review articles that have focused on nanoparticle engineering, system reconstruction techniques or general vascular and oncologic targeting instead of pulmonary imaging, there was a lack of attention to MPI. These studies give insights into tracer optimization and hardware development but do not provide much discussion on the physiological challenges in the lungs, such as air–tissue susceptibility interfaces, heterogeneity of pulmonary perfusion, respiratory motion and endothelial barrier dysfunction. The present review, however, offers a focused synthesis of pulmonary MPI applications, bringing together the physics of imaging, strategies for the design of tracers and biological models of the lungs in a common framework. In particular, we structure the existing literature according to the three major application areas of pulmonary imaging—perfusion imaging, therapeutic cell and aerosol tracking, and assessment of vascular permeability and inflammation—according to the studies and present validation strategies and quantitative imaging performance.

Furthermore, this review adds new topics that address the translational aspects of the iron oxide tracers, which are clinically approved, the combination of MRI with CT (MPI-CT), and the comparison with other clinically established pulmonary imaging methods, such as CTPA and ventilation–perfusion scintigraphy. This work brings together the technical, biological and translational viewpoints to create a pulmonary-centered synopsis, which complements and goes beyond previous MPI reviews.

MPI has shown great promise in pulmonary applications, including the ability to assess perfusion, and the ability to evaluate vascular health. With MAA-SPIONs, it is possible to quickly create three-dimensional perfusion maps which precisely depict the location of emboli causing perfusion deficits within minutes [5]. This characteristic can be a major help in making a diagnosis of an acute PE. Compromised capillary walls can be penetrated by custom SPION tracers as is done with Synomag. This allows us to determine the permeability of the vessels. It is an important indicator for the models of ALI, fibrosis and inflammation [6]. The recent findings suggest that MPI may be a valuable means of monitoring acute and chronic pulmonary diseases.

The improved diagnostic value of hybrid MPI-CT imaging technologies combines a number of the benefits of MPI with the full anatomical information provided by CT [8]. These two components together help to determine if there is a perfusion problem or vascular disruption in the context of the pulmonary architecture. Consequently, MPI-CT hybrid imaging may serve as a viable alternative to CTPA, as it does not involve hazardous radiation. This may be particularly beneficial for children, pregnant women, or individuals with renal issues who are concerned about excessive radiation exposure or contrast-induced nephropathy [58].

Before MPI may be widely exploited for clinical lung imaging, substantial technical and translational challenges must be addressed. Tracer pharmacokinetics must be enhanced to satisfy two distinct requirements: sufficient capillary retention for precise perfusion mapping and extended circulation durations for continuous, dynamic assessments [59]. Also, MPI systems have been successfully reduced to image small animals, but making the scanners bigger to fit the human chest demands special attention to the constraints of the particular absorption rate and peripheral nerve stimulation [60]. While preclinical pulmonary MPI studies are typically free from implant-related constraints due to the absence of intracardiac or pulmonary vascular devices in animal models, clinical translation must account for system-level safety considerations such as peripheral nerve stimulation and electromagnetic field dynamics, which constrain human MPI performance [55].

To employ MPI for lung imaging in a clinical context, a lot of experiments need to be done, including direct comparisons with well-known methods such as CTPA and V/Q scintigraphy. These studies must also account for patient-specific considerations, including appropriate tracer dosages for individuals with renal impairments and the establishment of safety and efficacy guidelines for regulatory approval. Furthermore, in order to be applied in real-life situations, it will be crucial to prove its effectiveness in the field beyond diagnosis to also increase speed, safety, and integration with current infrastructure [61].

The applications in new cancer therapy, such as magnetic hyperthermia, targeted drug delivery, and theranostics, are also receiving increased interest for the use of ferromagnetic nanoparticles. However, their wider biomedical use is restricted by magnetostatic attraction which tends to attract the cells together, which makes it difficult to stabilize them in the form of microscale aggregates. These interactions can affect the performance, magnetic response and repeatability of the biodistribution. Despite this, recent progress in surface functionalization, coating technologies and particle engineering still enhances the stability of nanoparticles and their growing potential for imaging and therapeutic uses.

Although MPI has many benefits, a number of limitations in the lung exist that hinder its clinical translation. The movement of the lung during respiration, the aeration heterogeneity, and the requirement for optimized tracer pharmacokinetics, with respect to the retention in the lung and the profile of systemic circulation, are still technically challenging in imaging the lung. Moreover, most MPI studies are conducted using custom-made SPION tracers that have not been approved for human use and the lack of human-scale MPI scanners further hinders the translation of MPI beyond preclinical settings. There is also variability in acquisition protocols, reconstruction strategies and tracer selection between studies, which further reduces comparability between studies. Overcoming these technical, regulatory and standardization hurdles will be crucial to the future of MPI from the experimental pulmonary imaging stage to clinical use.

## 8. Limitations

This review emphasizes that MPI is becoming a new tool for pulmonary imaging, but there are some significant concerns. First, most of the studies available are conducted in small animal models, mainly mice and rats, which have very different pulmonary physiology, respiratory motion and thoracic dimensions than humans. These discrepancies could have implications for direct clinical application of these tracers in terms of biodistribution, tracer sensitivity, and spatial resolution.

Second, many studies use experimental SPIONs like MAA-SPIONs, LS-008, Synomag and targeted molecular probes, which are not approved for clinical use. These agents have not been extensively studied in humans for their pharmacokinetics, safety or dosing regimens. Therefore, standardization of tracer formulations and regulatory approval paths are required prior to clinical implementation.

Third, currently available MPI systems focus mainly on imaging small animals, and implementing MPI hardware to image the human thorax is technically difficult, due to limitations on gradient strength, on peripheral nerve stimulation thresholds, and on maintaining acceptable spatial resolution over a wide field of view. In clinical applications these limitations can have an impact on the feasibility of imaging the whole lung.

Lastly, direct comparisons to other pulmonary imaging techniques like CTPA, MRI and V/Q scintigraphy are still sparse. A relative assessment of the diagnostic performance, cost effectiveness, and clinical utility of MPI cannot be made without head-to-head comparisons.

## 9. Conclusions

MPI is a fast-growing technology that could expand the field of functional lung imaging by directly imaging the distribution of the tracer and quantify pulmonary physiology. The ability of MPI to offer real-time imaging without the use of ionizing radiation gives it great potential in longitudinal imaging and, in the future, bedside use in pulmonary disease.

However, there are currently some problems that hinder clinical translation. Most studies of pulmonary MPI are limited to small animal models and variations in size, respiratory motion, and thoracic anatomy may impact performance in humans. Furthermore, most investigations use experimental SPION tracers not yet clinically approved, and standardized tracer pharmacokinetics and dosing strategies have yet to be defined. Other technical challenges in human-scale MPI scanner development, such as the requirement for strong gradients, low peripheral nerve stimulation thresholds and maintaining the spatial resolution within a large field of view, further limit clinical implementation. Furthermore, there are no direct comparison studies between established pulmonary imaging methods and MPI, which restricts evaluations of the diagnostic value of MPI.

Despite these restrictions, continuous development of tracer design, the scalability of scanners, motion compensation, and further hybrid MPI-CT or MPI-MRI integration will increase feasibility and expand applicability. Continued interdisciplinary collaboration between engineers, imaging scientists, and clinicians, along with prospective clinical validation studies, will be essential to translate experimental findings into clinical workflows. With further technical and regulatory progress, MPI may emerge as a complementary modality for functional and molecular pulmonary imaging, with potential roles in disease characterization, therapy monitoring, and precision medicine. For clarity, all abbreviations appearing in this manuscript are defined in Table 4.

## Figures and Tables

**Table 1:** Summary of key preclinical MPI studies on pulmonary perfusion and hemodynamic assessment.

Name	Year	Application	Experiment Type	Imaging Tracer	Conclusion
Zhou, Jeffris, Elaine, Zheng, Goodwill, Nahid and Conolly [5]	2017	Evaluation of MPI’s ability to assess rat lung perfusion using MAA-SPIONs and potentially diagnose PE.	In vivo and ex vivo	MAA-SPION	MAA-SPIONs were visible in MPI and assessed lung perfusion with high sensitivity, indicating MPI’s suitability for accurately and safely diagnosing life-threatening conditions like PE.
Kaul, Mummert, Jung, Salamon, Khandhar, Ferguson, Kemp, Ittrich, Krishnan and Adam [44]	2017	Evaluating the performance of a new tracer, LS-008, for MPI against the standard Resovist to improve diagnostic imaging.	In vitro and in vivo	LS-008 & Resovist	LS-008 significantly improves MPI with better image quality, clearer vascular delineation, and longer circulation times, enhancing clinical imaging.
Kaul, Mummert, Graeser, Salamon, Jung, Tahir, Ittrich, Adam and Peldschus [38]	2021	Noninvasive estimation of PBV in mice using a combined MPI–MRI approach.	In vivo	SPIONs	The combined MPI-MRI method accurately measured PBV in mice by tracking SPION bolus transit with MPI and obtaining cardiac volumetry with MRI, enabling reliable, noninvasive assessment of pulmonary hemodynamics.

**Table 2:** Preclinical studies demonstrating MPI for lung cell tracking and pulmonary therapeutic monitoring.

Name	Year	Application	Experiment Type	Imaging Tracer	Conclusion
Nigam, Uhl, Bamrah, Lin, Jager, Lawson, Girgis, Kenyon, Li and Li [46]	2025	Tracking transplanted distal lung epithelial progenitor cells in PF using MPI	In vivo and ex vivo	Dextran-coated iron oxide SPIONs	MPI successfully visualized and quantitatively tracked transplanted progenitor cells in fibrotic mouse lungs for 14 days, showing strong in vivo signal and ~65% signal decline over time, confirming reliable, noninvasive monitoring of cell retention and distribution in PF.
Tay, Chandrasekharan, Zhou, Yu, Zheng and Conolly [7]	2018	Tracking inhaled aerosol delivery, deposition, and clearance in the lungs using MPI	In vivo	SPION-labeled aerosol	MPI successfully visualized aerosol deposition, distribution, and mucociliary clearance from lungs to the GI tract. It enabled quantitative assessment of aerosol delivery efficiency and overcame MRI’s limitations, demonstrating MPI as a sensitive, non-radioactive method for monitoring pulmonary drug delivery.

**Table 3:** Preclinical MPI Studies Evaluating Lung Injury, Vascular Permeability, and Inflammation.

Name	Year	Application	Experiment Type	Imaging Tracer	Conclusion
Feng, Gao, Li, Hui, Jiang, Xie and Tian [6]	2024	Evaluation of the feasibility of using MPI signals to visualize and quantify pulmonary vascular leakage in both acute and chronic situations	In vivo	Dextran-coated SPION, Synomag^®^	3D MPI-CT successfully tracked pulmonary vascular disruption in both acutely injured and fibrotic mice lungs.
Feng [57]	2023	Quantitative assessment of pulmonary vascular permeability in ALI using MPI–CT	In vivo and ex vivo	Synomag-D SPIONs (3 mg/kg)	3D MPI-CT noninvasively visualized and quantified SPION extravasation in ARDS lungs, showing >2-fold increase in permeability compared to healthy mice. Quantitative MPI indices correlated with Evans Blue, lung wet/dry ratio, BALF protein levels, and histology, demonstrating MPI-CT as a reliable tool for monitoring vascular injury in vivo.
Gao, Liu, Wang, Feng, Liu, Liu, Huang, Wu, Xiong and Jia [52]	2024	Molecular-level imaging of intra-alveolar inflammation in ALI using VCAM-1-targeted MPI nanoprobe	In vitro and in vivo	VCAM-1-targeted polydopamine-coated SPION nanoprobe, ~10 nm	ESPVPN showed superior MPI performance, specific VCAM-1 targeting, and good biosafety. MPI signal strongly correlated with VCAM-1 expression and inflammatory biomarkers, enabling sensitive and quantitative detection of lung inflammation in ALI.

**Table 4:** Abbreviation table.

Abbreviation	Definition	Abbreviation	Definition
ALI	Acute Lung Injury	MRA	Magnetic Resonance Angiography
ARDS	Acute Respiratory Distress Syndrome	MRI	Magnetic Resonance Imaging
BALF	Broncho-Alveolar Lavage Fluid	OA	Oleic Acid
CKD	Chronic Kidney Disease	PBV	Pulmonary Blood Volume
CT	Computed Tomography	PE	Pulmonary Embolism
CTPA	CT Pulmonary Angiography	PF	Pulmonary Fibrosis
FFP	Field-Free Point	PTT	Pulmonary Transit Time
FFL	Field-Free Lines	SPECT	Single-Photon Emission Computed Tomography
HUVECs	Human Umbilical Vein Endothelial Cells	SEI	SPION Extravasation Index
LPS	Lipopolysaccharide	SPIONs	Superparamagnetic iron oxide nanoparticles
MPI	Magnetic Particle Imaging	VCAM-1	Vascular Cell Adhesion Molecule-1
MPS	Magnetic Particle Spectroscopy	V/Q	Ventilation/perfusion

## Data Availability

The paper contains all of the data.
